# Chronic Environmental and Occupational Lead Exposure and Kidney Function among African Americans: Dallas Lead Project II

**DOI:** 10.3390/ijerph15122875

**Published:** 2018-12-14

**Authors:** Robert Reilly, Susan Spalding, Brad Walsh, Jeanne Wainer, Sue Pickens, Marcene Royster, John Villanacci, Bert B. Little

**Affiliations:** 1 Nephrology Division, University of Alabama at Birmingham, Birmingham, AL 35233, USA; rreilly@uabmc.edu; 2 Medical Service, Birmingham VA Medical Center, Birmingham, AL 35233, USA; 3 Childrens Health Fund, New York, NY 10027, USA; sspalding@chfund.org; 4 Parkland Health and Hospital System, Dallas, TX 75235, USA; d.walsh@phhs.org (B.W.); jeanneew@att.net (J.W.); suepickens@att.net (S.P.); marcene_royster@yahoo.com (M.R.); 5 Environmental and Injury Epidemiology and Toxicology Branch, Texas Department of State Health Services, Austin, TX 78756 USA; jvilla@swbell.net; 6 Medical Service, VA North Texas Health Care System, Dallas, TX 75216, USA; 7 Department of Health Management and Systems Sciences, University of Louisville, Louisville, KY 40202, USA

**Keywords:** environmental factors, lead, renal function, African-Americans

## Abstract

*Background:* We examined the effects of lead on kidney function in occupationally and environmentally exposed adults from a Dallas lead smelter community that was the site of an Environmental Protection Agency (EPA) Superfund clean-up. All subjects were African Americans—a racial group that bears a disproportionate burden of kidney disease. *Methods*: A two-phase health screening was conducted. Phase II included a physical examination and laboratory tests. Study subjects were African Americans residents, aged ≥19 years to ≤89 years. Of 778 subjects, 726 were environmentally exposed and 52 were both occupationally and environmentally exposed. The effects of lead exposure on estimated glomerular filtration rate (eGFR) were examined in three groups: male and female smelter-community residents, as well as males with both occupational and environmental exposure. Multiple linear regression was used to analyze the dependence of eGFR on log (blood lead level), duration of residence in the community, type 2 diabetes, and hypertension. *Results*: There was a statistically significant negative effect on kidney function for all three groups. Comparison of female and male residents showed a slightly larger negative effect of blood lead level on eGFR in females versus males, with the largest effect seen in male smelter-working residents. For each unit increase (log_10_ 10 µg/dL = 1) in blood lead level, age-adjusted eGFR was reduced 21.2 mL/min/1.73 m^2^ in male residents, 25.3 mL/min/1.73 m^2^ in female residents and 59.2 mL/min/1.73 m^2^ in male smelter-working residents. *Conclusions:* Chronic lead exposure is associated with worsening kidney function in both African American male and female residents, as well as male workers in Dallas smelter communities. This effect is slightly, but not statistically significantly, worse in female residents than male residents, and significantly worse in males that both worked and resided in the smelter community.

## 1. Introduction

Lead is a classical environmental hazard, which has historical roots that date back several millennia. Long-term effects of environmental lead exposure have emerged as a serious health care concern over the past five decades. Lead pollution is a worldwide risk for all populations. Lead-associated renal disease is also an ancient association [1]. Nonetheless, controversy remains regarding the exact role and magnitude that chronic lead exposure plays in reduced kidney function [2,3]. Data on lead exposure and kidney function are drawn from studies of workers occupationally exposed, and suggest that lead is a cause of chronic kidney disease (CKD) [4,5,6]. Environmental lead exposure is also associated with diminished kidney function in subjects with no history of long-term occupational exposure. However, some investigators have failed to find a relationship between lead exposure and kidney function [7]. Alternatively, it was suggested that reduced kidney function itself may result in excessive lead accumulation [8]. Low level, long-term lead exposure may present non-specific clinical signs, or be subclinical and elude detection [9].

In the present investigation, estimated glomerular filtration rate (eGFR) is analyzed by sex among non-diabetic African-American adults, 19 to 89 years of age, who resided or worked in two lead smelter communities in Dallas, Texas. Glomerular filtration rate (GFR) is the most common measure used to assess overall kidney function. GFR can be measured directly using isotopes; however, this is cumbersome, costly, and involves radiation exposure. As a result, equations were developed to estimate it (eGFR), based on serum creatinine and other measurable factors (see Section 2.3: Estimated Glomerular Filtration Rate). Our study is important because it: (1) Assesses the effects of lead on kidney function in African Americans, a racial group that bears a disproportionate burden of kidney disease; (2) includes both occupationally and environmentally exposed subjects from the same community that was the site of an Environmental Protection Agency (EPA) Superfund clean-up; and (3) examines lead effects on kidney function separately, by sex.

## 2. Study Population and Methods

The present investigation was approved by the Institutional Review Board at University of Texas Southwestern Medical Center, Dallas, Texas (IRB#0902-495, 4 September 2002), that includes adherence to the declaration of Helsinki.

### 2.1. Population Background

In 1934, the first of three lead smelters was established in West Dallas, at the intersection of Singleton Boulevard and Westmoreland Street. Two additional smelters were opened, across the street from each other, in East Dallas, in the part of the city known as Cadillac Heights in the 1940s. After recognizing that environmental lead exposure was a public health problem in the 1960s, the city of Dallas initiated health surveys and established lead clinics to provide testing. Several clean-up projects that included soil removal were conducted over the years. Ultimately, blood lead levels decreased only when the smelters closed [10]. The companies were smelting lead from batteries and slag, releasing dust clouds over these communities. The West Dallas smelter was located near one of Dallas’ largest public housing projects. The last smelter closed in 1990, and the West Dallas area was designated an EPA Superfund Site [11]. New public housing was constructed within the old smelter community site.

### 2.2. Study Design

The study population is a convenience sample of people living in the postal codes when the smelters were in operation (1946–1984). About 2% of the population of postal code 75212, the site of the RSR smelter, participated in the study. Subjects were mainly African Americans (95.4%), reflecting the racial makeup of the exposed neighborhood in the 1970s. Twenty percent of subjects were age 65 or older, and the median age of subjects was 35, while the exposed population was slightly younger. Sixty-one percent of subjects were female. Therefore, the data are representative of the exposed population, are biased to inclusion of females, and are several years older than the 1970s–1980’s makeup of the West Dallas neighborhood. This suggests a greater window of environmental exposure to Pb in the neighborhood. Thus, the data are biased to include: (1) females, and (2) older individuals.

In 2002, a two-phase health screening was conducted in Dallas lead smelter communities. Phase I was a demographic and risk survey. Participants were recruited through town hall meetings and public service announcements in the media (newspapers, radio, and television). People who lived in the following zip codes were included: 75208, 75211, 75212, 75247, 75203, 75215, and 75216. The vast majority were from the West Dallas smelter community. Self-selected participants were invited to return for Phase II, which included a physical examination and clinical laboratory tests. All subjects in the present study were African American; 2% of the sample had race missing, or were listed as Hispanic, and were excluded. To be included in the analysis, subjects had to have: Values listed for age, sex, and race, in order to calculate an eGFR; an HbA1c value; and lived in a smelter area zip code. Subjects with diabetes mellitus (HbA1c ≥6.5%) were dummy coded (0,1) for regression analysis. Similarly, subjects with hypertension (blood pressure ≥140/90) were dummy coded (0,1) for regression analysis. Seven hundred and seventy-eight subjects met these criteria. Of these, 52 were smelter-working residents and 726 were non-smelter working residents. All of the included smelter-working residents were male. Fourteen female smelter-working residents were excluded from the analysis, because of missing data and small sample size.

### 2.3. Estimated Glomerular Filtration Rate (eGFR)

Estimated glomerular filtration rate (eGFR) was calculated using an ethnic and sex-specific four-variable MDRD (modification of diet in renal disease) equation: male eGFR = 186 × serum creatinine^−1.154^ × age ^−0.203^ × 1.21 and female eGFR = 186 × serum creatinine^−1.154^ × age ^−0.203^ × 1.21 × 0.742 [12]. We used the four-variable MDRD equation in order to compare our results to previous studies which also used this equation. Blood lead level was log_10_ transformed, because the variable was right skewed (>1.0). Log linear regression was used to analyze eGFR by sex, controlling for age-related decline in glomerular filtration rate (GFR), given that the ages of the groups were different and GFR declines with age.

### 2.4. Analysis

Multiple linear regression was used to analyze the dependence of eGFR on log (blood lead level), duration of residence in the community, type 2 diabetes, and hypertension. The regression formula used was: eGFR = log_10_(Blood Lead, µg/dL) + Duration of Residence (years) + Type 2 Diabetes (0,1) + Hypertension (0,1) + Smoking Tobacco (0,1) + Intercept, where 0 = No, 1 = Yes.

### 2.5. Software

SAS (v.9.1, SAS Institute, Cary, NC, USA) and SPSS (v.18, IBM, Chicago, IL, USA) were used to analyze the data.

## 3. Results

Study group characteristics are shown in Table 1. Data were analyzed separately by sex. Male smelter-working residents, on average, were older than non-smelter working residents since they were employed at the time of the smelter operation, while many of the residents were children and adolescents. The average number of years residing in the lead smelter community was comparable between the three groups. Mean and interquartile values are also shown for blood lead level, serum creatinine concentration, and eGFR in Table 1. Blood lead levels were highest in male smelter-working residents, followed by female residents. Male residents had the lowest blood lead levels of the three groups.

Regression of eGFR on log blood lead level in all residents showed a statistically significant decline (B = −22.84, *p* = 0.0001). Regression of eGFR on log blood lead level in male residents showed a statistically significant decline (B = −21.19, *p* = 0.001). For each unit (log_10_ 10 µg/dL = 1) increase in blood lead level, eGFR declined by 21.2 mL/min/1.73 m^2^ (Table 2). In female residents, the effect was larger (B = −25.27, *p* = 0.0001). For each unit (log_10_ 10 µg/dL = 1) increase in blood lead level, eGFR declined by 25.3 mL/min/1.73 m^2^ (*p* = 0.001). The greatest effect of all was seen in male smelter-working residents (B = −59.16, *p* = 0.0001). For each unit (log_10_ 10 µg/dL = 1) increase in blood lead level, eGFR was reduced 59.2 mL/min/1.73 m^2^. The lion’s share of reduced eGFR in smelter-working residents was related to the detrimental effects of lead on the kidney.

The regression slopes for male and female residents were within 95% confidence intervals (CI) of one another (males: −7.19 to −35.19; females: −15.33 to −35.21), and are therefore not significantly different. However, the Pb slope for male smelter workers is significantly higher than male and female residents, with a 95% CI of −85.89 to −32.43.

## 4. Discussion

Most previous studies have reported an association between environmental lead exposure and worsening kidney function in the United States, Belgium, the United Kingdom, and Taiwan. A selected sample of these are depicted in Supplementary Materials Table S1 [13,14,15,16,17,18,19,20,21,22]. The environmental exposure reported was primarily that of living in the country of origin, with no known additional risk. For example, the majority of studies examining this issue in the United States come from the National Health and Nutrition Examination Surveys (NHANES) and the Normative Aging Study. NHANES studies were carried out in a random selection of United States residents, and the Normative Aging Study in a predominantly Caucasian male veteran population in Boston (100% male, 97% Caucasian), where the primary mode of lead exposure was likely from leaded gasoline and paint. Our study is unique, because our subjects lived in a lead smelter community that was the subject of an EPA Superfund clean-up, and were exposed to a heavy atmospheric lead burden. Importantly, we include both occupational and environmentally exposed subjects from the same community.

Severity of exposure is illustrated by lead levels documented in residents of this community over time [10,23,24]. Mean blood lead levels in children and young adults in the 1980s, prior to closure of the smelters and the EPA Superfund clean-up, ranged from 15.8–24.8 µg/dL, with values as high as 77 µg/dL. In later years, lead levels fell to a range of 1.8–3.7 µg/dL, after the smelters closed and clean-up was completed. Despite the limitation that blood lead levels are a better indicator of short term (rather than cumulative) lead exposure, they remain the standard for lead exposure given the difficulty of readily obtaining bone biopsies in large groups of subjects [9]. It is important to point out that, in our study, blood lead levels were measured in 2002 and may not be an accurate reflection of lead levels across a smelter community resident’s lifetime. However, given blood lead levels observed in this community in the past, it is highly likely that adult residents had blood lead levels of 20 µg/dL or greater as children and adolescents.

In 1984, the city of Dallas initiated a clean-up in West Dallas. Soil was replaced on 40 acres of land within a quarter mile of the smelter; sod and grass were planted in bare spots, unpaved driveways, and alleyways. In 1993, the area was designated an EPA Superfund clean-up site. Soil was removed in residential areas with a lead level >500 ppm in 413 residential lots, two schools, two churches, and three sites where battery slag was dumped. At the smelter site itself, 700 cubic yards of hazardous soil, 600 drums of waste material, 90 waste debris piles, and 1700 gallons of hazardous liquids were removed. The soil content of lead near the smelter was, in many cases, >5000 ppm.

We examined the effects of blood lead level on eGFR in men and women separately. The observed effect of a reduction in eGFR of 59.2 mL/min/1.73 m^2^ in male smelter residents is higher than that previously reported by Payton in the United States [17] and Staessen in Belgium [14]. In the Cadmibel study from Belgium, the effect on creatinine clearance was also larger in women than men [14]. One possible explanation for a slightly larger lead-related effect on eGFR in female smelter residents is increased lead release from bone [25]. Lead can deposit in multiple organs, including kidney, teeth, liver, and bone. In the long term, most of the total body lead burden (95%) is in bone. Higher bone turnover and release of stored lead may explain part of the increased toxicity in female smelter residents. Alternatively, female residents may have had more exposure to the local smelter environs, since, during this era, women spent more time in the home, whereas male residents may have worked outside the community. In addition, blood lead level was higher in female residents than male residents, in contrast to other studies that reported lead levels by sex, where blood lead levels were higher in males compared to females [14,15,16,21].

We also examined the effect of lead exposure on eGFR in residents and workers in the community. Occupational lead exposure is associated, in most [26,27,28,29,30,31] but not all [32,33] studies, with increased mortality from renal-related causes. Studies of occupational lead exposure on renal function have yielded conflicting results [7,34,35,36,37,38,39,40,41,42,43,44,45,46], and a selected group is shown in Supplementary Materials Table S2. Two reports controlled for effects of age-related decline in GFR [41,44]. One study was made up predominantly of black battery factor workers in South Africa [44]. Control groups varied from none at all to nonexposed workers in the same factories, nonexposed workers in other factories, postal workers, other industry workers, or residents living in distant rural locations. Our study showed a greater effect of lead on renal function in smelter-working residents. This is likely due to increased lead exposure, in that they both worked and resided in the smelter community, and is supported by the fact that, in 2002, they also had the highest blood lead levels of the three groups.

One weakness of our study is that it is cross sectional. We did not have data over time and, as a result, our findings are associative in nature. One could make an argument for reverse causation in that the GFR decline could result in total body lead accumulation, rather than the reverse explanation that lead injures the kidney and results in a decline in GFR. At least one previous study argues against this [47]. In patients with end-stage renal disease (ESRD) that underwent transiliac bone biopsy, renal failure itself did not increase bone lead content. Therefore, it is more likely that chronic lead exposure results in reductions in GFR and increased risk of ESRD and CKD, rather than the reverse (decreased GFR causes lead accumulation). Another limitation is that all of our subjects were African American, which limits generalizability to other racial groups. However, African Americans are 3–4 times more likely to progress to ESRD than Caucasians [48]. African Americans make up about one third of all ESRD patients, despite the fact that they make up about only about 12% of the United States population. This is likely the result of a combination of environmental, genetic, and socioeconomic factors. Some may argue that, with removal of lead from gasoline and paint, and with the closure of the last remaining smelter in the U.S. in 2013, that environmental lead exposure is no longer an issue in this country. Unfortunately, this is not the case. Recently, drinking water has emerged as a source of lead. It was not until 1986, when the Safe Drinking Water Amendments were passed, that installation of pipes containing lead was prohibited [49]. However, in many cities, lead pipes serve as feeder lines, connecting water mains to older buildings. The addition of ferric chloride to reduce organic matter in the Flint River increased the corrosivity of the water, and liberated lead from feeder lines [50]. From 2013 to 2015, the number of children in Flint whose lead levels exceeded 5 µg/dL rose from 2.4% to 4.9% [51].

The effects of cigarette smoking on GFR are conflicting, and were nicely summarized in a review by Noborisaka [52]. At the time the manuscript was published (2012), five studies detected a decline in GFR, while nine reported an increase. One study showed elevated risk for both an increased and decreased GFR in the same study population [53]. Yoon, in a report of apparently healthy Koreans, found that smoking was associated with an increase in eGFR in those with an eGFR ≥ 50 mL/min; however, smoking was associated with decreased eGFR in those with a GFR < 50 mL/min [54]. This data suggests that there may be a subset of the population that is susceptible to renal injury, secondary to smoking. An increase in GFR may be due to at least two potential mechanisms. Smokers and nonsmokers respond differently to the effects of nicotine [55]. Smokers show an increase in urinary cyclic guanosine monophosphate, which is generally associated with renal vasodilation. This raises the possibility that smokers and nonsmokers may have different intrarenal hemodynamic responses to nicotine, resulting in vasodilation and an increased GFR in some smokers. Chronic cigarette smokers were also reported to be hyperinsulinemic [56]. Administration of insulin to normal individuals increases GFR [57].

## 5. Conclusions

In conclusion, the results of our current study show that chronic lead exposure is associated with worsening kidney function in both African American male and female residents, and male workers residing in Dallas smelter communities. This association is slightly but not significantly greater in female residents than in male residents, and is significantly more pronounced in those that both worked in the smelter and lived in the community.

## Figures and Tables

**Table 1:** Study group characteristics.

Interquartile Range					
Total Sample	*N*	Mean	SD	25th	75th
Age (years)	778	46.9	14.5	36	57
Duration of residence (years)		19.4	13.4	9	27
Blood Lead (µg/dL)		2.2	2.2	1	3
Creatinine (mg/dL)		1.04	0.53	0.9	1.1
eGFR (mL/min/1.73 m^2^)		91.4	24.1	76.4	105.4
Diabetes **	117	15.00%			
Hypertension ***	568	73.00%			
Smoke Tobacco	230	29.50%			
**Male, Resident:**					
Age (years)	290	43	14.1	33	48.8
Duration of residence (years)		11.5	11.9	0	24
Blood Lead (µg/dL)		2.7	2.5	1	3
Creatinine (mg/dL)		1.2	0.66	1	1.2
eGFR (mL/min/1.73 m^2^)		96	24.20%	84.3	108.7
Diabetes **	23	7.90%			
Hypertension ***	172	59.30%			
Smoke Tobacco	104	36.00%			
**Female, Resident:**					
Age (years)	436	47.7	14.3	25.3	54
Duration of residence (years)		13.1	12.9	10	24
Blood Lead (µg/dL)		1.8	1.4	1	2
Creatinine (mg/dL)		0.94	0.41	1	1.2
eGFR (mL/min/1.73 m^2^)		89.8	23.3	76.5	102.2
Diabetes **	82	18.80%			
Hypertension ***	356	81.70%			
Smoke Tobacco	104	23.80%			
**Male, Smelter-working resident:**					
Age (years)	52	55.8	10.5	48	64
Duration of residence (years)		14.1	12.2	7.3	30.8
Blood Lead (µg/dL)		4.5	5	2	5
Creatinine (mg/dL)		1.3	0.67	1	1.4
eGFR (mL/min/1.73 m^2^)		85.2	26.5	68.5	103.2
Diabetes **	12	23.10%			
Hypertension ***	40	76.90%			
Smoke Tobacco	28	53.80%			

All subjects are African American; ** HbA1c ≥ 6.5; *** 140/90 mmHg or greater, or on anti-hypertensive medications. eGFR: Estimated glomerular filtration rate.

**Table 2:** Regressions of estimated glomerular filtration rate (eGFR) on log_10_ blood lead, duration of residence, diabetes, and hypertension: Effects on Renal Function.

GFR Units Scaled	*N*	B	SE	*p*	eGFR Decrement per 10 μg/dL ↑ Pb
**Total Sample**					
Log blood lead	778	−22.84	3.85	0.0001	−22.8 mL/min/1.73 m^2^
Duration of residence		−0.11	0.07	0.11	
Diabetes		−7.42	2.42	0.002	
Hypertension		−8.08	1.9	0.0001	
Smoking Tobacco		7.9	0.99	0.0001	
Intercept		107.67	2.29	0.0001	
**Male: Resident**					
Log blood lead	290	−21.19	7.15	0.003	−21.2 mL/min/1.73 m^2^
Duration of residence		0.04	0.13	0.75	
Diabetes		−1.36	5.47	0.8	
Hypertension		−5.87	3.47	0.09	
Smoking Tobacco		9.95	3.51	0.005	
Intercept		106.39	4.47	0.0001	
**Female: Resident**					
Log blood lead	436	−25.27	5.07	0.0001	−25.3 mL/min/1.73 m^2^
Duration of residence		−0.10	0.08	0.22	
Diabetes		−8.40	2.82	0.003	
Hypertension		−9.46	2.29	0.0001	
Smoking Tobacco		4.22	2.59	0.11	
Intercept		107.83	2.74	0.0001	
**Male: Smelter Worker**					
Log blood lead	52	−59.16	13.64	0.0001	−59.2 mL/min/1.73 m^2^
Duration of residence		−0.54	0.23	0.03	
Diabetes		−9.09	7.98	0.26	
Hypertension		−0.90	9.16	0.92	
Smoke Tobacco		13.11	7.18	0.08	
Intercept		133.98	15.14	0.0001	

B—regression slope; SE—standard error.

## Supplementary Materials

The following are available online at http://www.mdpi.com/1660-4601/15/12/2875/s1, Table S1: Selected studies of the effects of lead exposure on kidney function. Table S2: Selected studies of the effects of occupational lead exposure on kidney function.
