# Supplementary material for: Chronic Environmental and Occupational Lead Exposure and Kidney Function among African Americans: Dallas Lead Project II
Source: Int J Environ Res Public Health. 2018 Dec 14;15(12):2875. doi: 10.3390/ijerph15122875 (PMC6313544; doi:10.3390/ijerph15122875)
Supplement: Supplementary file 1 [file ijerph-15-02875-s001.pdf]

Supplementary Table S1. Selected studies of the effects of lead exposure on kidney function.

Author	Population	Study	Blood lead levels-mean	Effect
Muntner [13] 2003	NHANES III- USA 1988–1994	Cross-sectional *N*= 15,211	4.21 µg/dL- HTH<br>3.3 µg/dL- no HTN	Comparing highest to lowest quartile of lead levels OR of increased creatinine 2.41 and of CKD 2.60 in those with hypertension, OR increased in men and women, in AA OR increased for CKD but not increased creatinine, no effect in normotensives
Staessen [14] 1992	Belgium- Cadmibel study 1985–1989	Cross-sectional *N*= 1,981	11.4 µg /dL- men<br>7.5 µg/dL- women	Every 10 fold increase in blood lead levels was associated with a 10–13 ml/min decrease in creatinine clearance. Measured creatinine clearance was decreased by 10 mL/min in men and 13 mL/min in women. Calculated creatinine clearance was reduced 13 mL/min in men and 30 mL/min in women.
Munter [15] 2005	NHANES III and NHANES 1999–2002-USA	Cross-sectional *N*= 26,570	2.76 µg/dL- NHANES III<br>1.64 µg/dL- 1999-2002<br>Higher in men	Comparing highest to lowest quartile of lead levels OR of CKD 1.92
Staessen [16] 1990	London- civil servants	Cross-sectional *N*= 531	11.4 µg /dL- men<br>9.6 µg/dL- women	In men for each 25% increase in blood lead level the serum creatinine increased 0.6 mmol/L (NS), no effect on serum creatinine in women, no effect on blood pressure
Payton [17] 1994	Normative Aging Study VA 1988–1991	Cross-sectional *N*= 774 men	8.1 µg/dL	Every 10.0 µg/dL rise in blood lead level was associated with a 10.4 mL/min decrease in creatinine clearance
Kim [18] 1996	Normative Aging Study VA 1979–1994	Retrospective cohort 459 men	9.9 µg/dL	A 10-fold increase in blood lead levels was associated with a 0.08 mg/dL increase in serum creatinine concentration
Tsaih [19] 2004	Normative Aging Study VA 1991–1995	Prospective 448 men	6.5 µg/dL	Increased rate of rise of serum creatinine in the highest versus lowest quartile of blood lead levels that was more pronounced in those with diabetes.
Navas-Acien [20] 2009	NHANES 1999- 2006-USA	Cross-sectional *N*= 14,778	1.58 µg/dL	Comparing highest to lowest quartile of blood lead levels OR of CKD 1.56
Spector [21] 2011	NHANES 1999–2002 USA	Cross-sectional *N*= 3,941	2.1 µg/dL- men<br>1.4 µg/dL- women	In patients aged ≥60 comparing highest to lowest tertile of blood lead levels there was a 7.1 mL/min/1.73 m^2 decline in eGFR. Each doubling of lead level was associated with a 3.3 mL/min/1.73 m^2 decline in eGFR

Yu [22] 2004	Taiwan	Prospective- 4 yrs 121 CKD patients	4.2 µg/dL	Each 1.0 µg/dL increase in blood lead level reduced the GFR by 4 mL/min

Abbreviations: VA- Veteran's Affairs, NHANES- National Health and Nutrition Examination Survey, OR- odds ratio, CKD- chronic kidney disease, AA- African Americans, NS- not significant.

Supplementary Table S2. Selected studies of the effects of occupational lead exposure on kidney function.

Author	Country	Mean lead level (μg/dL)	N	Exposure	Effect
Gennart [34] 1992	Belgium	51.0	81	Battery factory<br>Controls- 81 nonexposed workers in the finishing and maintenance department of the same factory, hospital warehouse, chemical factory	No renal effects
Weaver [35] 2003	Korea	32.0	803	Many different lead industries<br>Controls- 98 air conditioner assembly plant and university employees	Association with increased BUN
Oktem [36] 2004	Turkey	7.79	79	Auto repair shop<br>Controls- 71 people living in a rural area	No effect on GFR
Gerhardsson [37] 1992	Sweden	31.9 (median)	100	Smelter<br>Control- 41 truck assembly workers with no lead exposure living more than 140 km from the smelter	Urinary albumin, β2-microglobulin and NAG increased, no change in creatinine
Baker [38] 1979	USA	16–280 (range)	160	Two smelters, chemical plant<br>No controls	8 subjects with elevated creatinine
de Almeida [39] 1987	Brazil	64.1	52	Smelter<br>Controls- 44 paper mill workers in the same city	32.7% with creatinine >1.5 mg/dL
Chia [40] 1995	Singapore	NA	137	Lead stabilizer<br>153 postal workers with no history of lead exposure	8 with decreased creatinine clearance, elevated urine β2-microglobulin
Greenberg [41] 1986	USA	32	38	Enamel paint<br>No controls	Creatinine clearance normal, 2 with proteinuria and 3 with elevated urine β2-microglobulin
Lilis [42] 1980	USA	NA	269	High level exposure- lead smelter low level exposure- cable manufacturers, cable splicers, firearms instructors, no controls	Lead levels correlated with BUN and urine β2 microglobulin, 18% creatinine >1.2 mg/dL, 8% creatinine >1.4 mg/dL, 20 workers with >20 years exposure 45% Had serum creatinine >1.4 mg/dL
Verschoor [43] 1980	Netherlands	47.5	155	Lead industry workers<br>Controls- insulation, drain pipe and concrete workers with no lead exposure	Increased urine retinol binding protein and NAG
Buchet [44] 1980	Belgium	44.3	25	Smelter<br>Controls- judged to be nonexposed by plant doctor	No renal effects
Ehrlich [45] 1998	South Africa	53.5	382	Battery factory<br>No controls	Lead levels correlated with serum creatinine
dos Santos [46] 1994	Brazil	36.8	166	Battery factory<br>Controls- 60 nonexposed workers	Increased urine NAG, no albuminuria, no increase in creatinine
Omae [47] 1990	Japan	36.5	165	Lead storage battery manufacturing plant<br>No controls	No renal effects

Abbreviations: km- kilometers, BUN- blood urea nitrogen, GFR- glomerular filtration rate, NAG-N-acetyl--β-glucosaminidase.
